# Peer Review of “Advancing Early Detection of Major Depressive Disorder Using Multisite Functional Magnetic Resonance Imaging Data: Comparative Analysis of AI Models”

**DOI:** 10.2196/76744

**Published:** 2025-07-15

**Authors:** ­ Anonymous

**Keywords:** major depressive disorder, machine learning, functional MRI, early detection, artificial intelligence, psychiatry

*This is a peer-review report for “Advancing Early Detection of Major Depressive Disorder Using Multisite Functional Magnetic Resonance Imaging Data: Comparative Analysis of AI Models.”*

## Round 1 Review

 This paper [1] addresses a relevant and important topic in psychiatric research. The authors aim to develop and compare machine learning models for early detection of major depressive disorder using functional magnetic resonance imaging (fMRI) data, which is a novel and promising approach. The study appears to be well structured and utilizes an appropriate set of methodologies to evaluate the machine learning models. However, some issues need to be addressed before the manuscript can be considered for publication.

### Specific Comments

#### Major Comments

- Interpretability of artificial intelligence (AI) models: While the paper discusses the models’ performance, it would benefit from further elaboration on the interpretability of the models, particularly the clinical relevance of Shapley additive explanations values and activation maximization findings. Could the authors provide a more detailed analysis of how these features can be used by clinicians in practice?
- Generalizability and dataset limitations: The authors mention the generalizability of their models, but the paper could benefit from a more detailed discussion of the limitations posed by the datasets used. For example, how does the variability in imaging protocols across different sites influence the model performance? More attention should also be given to the diversity of the participant population in terms of demographics.
- Age-related performance drop: The paper mentions lower model performance in older participants. This is a significant finding and should be explored further. Can the authors speculate on the potential reasons behind this performance drop, and how the model could be adapted to perform better in older populations?

#### Minor Comments

- Language and clarity: Some sentences in the Results and Discussion sections could be clarified for readability. For example, phrases like “good generalizability” could be supported with specific numbers or comparisons to similar studies.
- Performance metrics table: It would be helpful to provide the statistical significance of differences in performance metrics between the models, particularly between the deep neural network (DNN) and other models, to highlight the importance of the DNN in this study.
- Ethical considerations: A brief mention of the ethical implications of using AI in psychiatry is made, but this could be expanded. Ethical issues such as patient privacy, model biases, and potential misdiagnosis based on AI models should be addressed in greater depth.

## Round 2 Review

The paper presents an analysis of several AI models (support vector machine, random forest, gradient boosting machine, and DNN) for the early detection of major depression disorder using multisite fMRI data. The study offers valuable insights into both predictive performance and model interpretability. It is commendable that the authors leverage a diverse dataset and employ robust validation techniques (eg, 5-fold cross-validation and external validation) to assess model generalizability. However, there are areas—particularly in methodological clarity and discussion of clinical translation—that would benefit from further refinement.

### Major Comments

#### Methodological Details and Preprocessing

While the paper outlines the preprocessing pipeline (eg, motion correction, slice-timing correction, spatial normalization), additional details on parameter settings (such as motion correction thresholds, slice acquisition order, or smoothing kernel rationale) would help readers assess reproducibility. Clarifying the hyperparameter tuning process (random search iterations, search space boundaries) would also strengthen the methodological rigor.

#### Data Heterogeneity and Generalizability

The study uses fMRI data from three public datasets, which is a strength in terms of diversity. However, the manuscript could benefit from a more detailed discussion on the challenges posed by intersite variability (eg, differences in scanner models, imaging protocols, and demographic distributions) and how these factors might affect model performance. Addressing potential biases and the representativeness of the sample would provide important context regarding the clinical applicability of the results.

#### Interpretability and Clinical Integration

The inclusion of feature importance and Shapley additive explanations analyses is a positive step toward interpretability. Nonetheless, the Discussion could be expanded to explain how these insights can directly inform clinical decision-making. For example, a deeper exploration of how the identified neural connectivity patterns relate to established neurobiological theories of major depressive disorder—and what this means for potential treatment interventions—would enhance the translational impact of the work.

### Minor Comments

#### Clarity and Language

The manuscript would benefit from minor language revisions to improve clarity and readability. Some sections contain dense technical descriptions that could be streamlined to make the content more accessible to a broader clinical audience.

#### Figures and Tables

Ensure that all figures (especially the model performance comparison chart) and tables are clearly labeled and of sufficient resolution. Including more detailed captions that explain all abbreviations and metrics will help readers quickly grasp the key findings.

#### Discussion Section

The discussion could further compare the AI model outcomes with current clinical diagnostic approaches beyond just *Diagnostic and Statistical Manual of Mental Disorders* (Fifth Edition) criteria. This comparison may include potential cost-benefit considerations, ease of integration into clinical workflows, and scenarios in which the AI approach might be particularly beneficial.

#### Future Directions

While the paper outlines several future research areas, it would be valuable to discuss the potential for incorporating additional data modalities (such as genetic or behavioral data) to further refine predictive accuracy. Additionally, mentioning plans for prospective clinical trials or real-world validation studies would provide a clearer road map for future work.

## Abbreviations

AI: artificial intelligence
DNN: deep neural network
fMRI: functional magnetic resonance imaging
